# Formal definition of the MARS method for quantifying the unique target class discoveries of selected machine classifiers

**DOI:** 10.12688/f1000research.110567.1

**Published:** 2022-04-04

**Authors:** Felipe Restrepo, Namrata Mali, Alan Abrahams, Peter Ractham

**Affiliations:** 1 Department of Industrial and Systems Engineering Information,, Virginia Tech, Virgina, 24061, USA; 2 Department of Computer Science, Virginia Tech, Virginia, 24061, USA; 3 Department of Business Information Technology, Virginia Tech, Virginia, 24061, USA; 4 Department of Management Information Systems, Thammasat University, Bangkok, 10200, Thailand

**Keywords:** Machine learning, Binary classification, Classifier performance evaluation, Classifier selection optimization, Classifier comparative uniqueness

## Abstract

Conventional binary classification performance metrics evaluate either general measures (accuracy, F score) or specific aspects (precision, recall) of a model’s classifying ability. As such, these metrics, derived from the model’s confusion matrix, provide crucial insight regarding classifier-data interactions. However, modern- day computational capabilities have allowed for the creation of increasingly complex models that share nearly identical classification performance. While traditional performance metrics remain as essential indicators of a classifier’s individual capabilities, their ability to differentiate between models is limited. In this paper, we present the methodology for MARS (Method for Assessing Relative Sensitivity/ Specificity) ShineThrough and MARS Occlusion scores, two novel binary classification performance metrics, designed to quantify the distinctiveness of a classifier’s predictive successes and failures, relative to alternative classifiers. Being able to quantitatively express classifier uniqueness adds a novel classifier-classifier layer to the process of model evaluation and could improve ensemble model-selection decision making. By calculating both conventional performance measures, and proposed MARS metrics for a simple classifier prediction dataset, we demonstrate that the proposed metrics’ informational strengths synergize well with those of traditional metrics, delivering insight complementary to that of conventional metrics.

## Introduction

Traditionally, binary classification performance has been assessed using a combination of statistical measures derived from the classifier’s confusion matrix (accuracy, precision, recall/sensitivity, specificity, F score), or the classifier’s various confusion matrices, in the case of classifications at different cut-off thresholds (ROC curve, AUC metric). Accuracy is defined as the percentage of correct predictions out of all predictions. Precision is the percentage of predicted positives that are true. Recall (sensitivity) is the percentage of actual positives that are correctly predicted. Specificity is the percentage of actual negatives that are correctly predicted. F scores (various variants like F _1_, F _2_) combine precision and recall, weighting each equally, or unequally, to account for different misclassification costs. Finally, for binary classifiers that assign a probability or score to predictions, ROC curves and AUC metrics account for these ranked predictions, allowing for sensitivity and specificity to be observed at different cut-off thresholds. To plot the ROC curve and assess AUC, sensitivity and specificity are measured @k, where k is the number of top-ranked predictions and increases from 1 to the total number of observations in the dataset. Effective classifiers demonstrate a “bulge” in the ROC curves, and concomitant AUC close to 1, indicating that they discover far more true positives in the top-ranked k items, than would be expected in a random selection of k items. Notably, none of these conventional metrics assess the distinctiveness (uniqueness) of the classifier’s predictions, relative to other classifiers. In other words, conventional metrics are unable to assess what percentage of true positives (‘hits’) are found only by the current algorithm but not by alternatives, nor what percentage of false negatives (‘misses’) were missed by the current algorithm but not by alternatives. The inability of conventional classifier evaluation metrics to quantify how many, and what proportion, of a classifier’s correct (and incorrect) predictions are exclusive to that classifier, is a significant limitation. Two classifiers of equal accuracy (or precision, or recall, or AUC) may each have the unique ability to identify distinct observations from the target class, and this classifier uniqueness ought to be assessable.

Such assessments about classifier uniqueness have been made possible through the use of novel MARS ShineThrough and MARS Occlusions scores, whose software-level implementation was recently described in Ref. 25. However, since ^25^ focuses solely on the usage and interpretation of the software artifact’s outputs, it does not outline the methodological framework used to generate ShineThrough and Occlusion scores. Hence, in this paper, we present the mathematical foundations behind MARS metrics and their corresponding software artifact. Furthermore, we also provide step-by-step sample calculations that illustrate the inner workings of Shinethrough and Occlusion scores for a simple dataset. Being able to quantitatively assess classifier uniqueness has multiple benefits: better decisions could be made about combining complementary classifiers (vs duplicative classifiers), and improved characterizations could be run of where particular classifiers ‘shine through’ (spot true positives that no other classifiers spot) or ‘occlude’ (hide or miss observations in the target class, by mistakenly classifying those observations as false negatives, when one or more of the other classifiers have been able to spot those observations as true positives).

As an example of the problematic omission of exclusivity metrics in the evaluation and comparison of classifiers, consider the following cases. Recently, ^1^ evaluated the generalized, binary predictive ability of eight classifiers across ten datasets. ROC curve values for the top-ranked classifiers revealed that Support Vector Machine (SVM), Artificial Neural Network (ANN), and Partial Least Squares Regression (PLS) classifier performances were nearly identical across all datasets. ^2^ compared the performance of several classifiers, namely, Random Forest (RF), Decision Tree (DT), and k-nearest neighbors (kNN), using binary classification schemes for variable stars. Similar to Refs. 1, 2’s precision, recall, and F _1_ scores indicated that all three classifiers performed nearly identically. ^3^^–^^5^ reported similar outcomes, with virtually equal performance metric values across the top n-ranked classifiers. In all these cases, while the performance of the classifiers is nearly identical according to conventional classifier evaluation metrics, the classifiers clearly made different false positive and false negative errors, and thus triumphed, or failed, relative to other classifiers on particular observations. Clearly, the scope of traditional statistical performance measures is too narrow to provide the insight required to distinguish between the top n-ranked classifiers based on their respective exclusive hits or misses. Regardless of classifier ranking, traditional performance metrics, such as accuracy and F _1_ score, may not reliably reflect the classifier’s true performance, particularly on imbalanced datasets. ^6^ Novel classifier exclusivity metrics are needed to illustrate the success or failure of classifiers on particular observations, relative to their competing classifiers. These exclusivity metrics should reflect the extent to which a classifier exclusively finds (“shines through”) observations in the target class (that are not spotted by competing classifiers), or exclusively misses (“occludes”) observations in the target class (that are spotted by competing classifiers).

Consider a classification task where the data scientist is attempting to identify safety concerns expressed by consumers in millions of online product reviews (e.g., see Refs. 7– 10), using alternative candidate classifiers C _1_ and C _2_. The classification task is critical: missed safety concerns are unaddressed product hazards that could injure current or future product users. Assume the two competing classifiers, C _1_ and C _2_, both have precision of 80%, and recall of 80%, superficially (i.e., prima facie) indicating the classifiers have similar performance. However, if we are able to take into consideration the exclusivity of the classifier’s predictions (“shine through” and “occlusion”), we may find that C _1_ finds a significant proportion of the target class (safety concerns, in this observation) that C _2_ misses (“occludes”). Assessing classifier exclusivity is thus essential to revealing that two classifiers with 80% precision are by no means identical in their target- observation discovery ability, and may be complementary, rather than simply competing. This realization allows the data scientist to discover more safety concerns, through intelligent classifier combination (e.g., taking true positives from both classifiers), rather than the data scientist simply deciding to eliminate a superficially comparable classifier (when regarding conventional classifier performance metrics only prima facie).

Due to conventional metrics’ vulnerability to class imbalance, researchers have sometimes adopted alternative performance measures that complement traditional classifier evaluation techniques and help provide a more accurate assessment of the classifier’s true performance. Commonly used alternative measures include Cohen’s kappa ^11^ and Matthews Correlation Coefficient (MCC). ^12^ Cohen’s kappa (1960) calculates the agreement between the model’s predicted class labels and the actual class labels. Multiple studies ^6^^,^^13^^,^^14^ have identified concerns, relating to interpretability and class imbalance, when using Cohen’s kappa for binary classification. Regarding interpretability, the use of a relative metric (Cohen’s kappa) to evaluate model performance may lead to inconsistent results in which superior classifiers receive low kappa scores. ^14^ Additionally, imbalanced class labels generally produce higher kappa scores, generating overoptimistic results that do not reflect true model performance. ^14^ MCC, generally used in imbalanced classification, relies on all four confusion matrix categories (true positives, true negatives, false positives, and false negatives) and is invariant to class label distributions, thus, yielding scores that better assess imbalanced classification performance. ^6^ While the class imbalance problem has received significant attention, the identification and quantification of a classifier’s prediction exclusivity (distinctive predictive successes and failures relative to competing classifiers) has not been studied.

Current conventional and alternative classifier performance metrics suggest that the behavior of elite models is generally indistinguishable from that of other elite models. Nevertheless, fundamentally differing mathematical and structural assumptions between different classifier algorithms indicate otherwise, implying that successful classifiers may not be as similar to each other as suggested by current metrics.

In this paper, we present the methodology for MARS (“Method for Assessing Relative Sensitivity/Specificity”), a novel approach that evaluates the comparative uniqueness of a classifier’s predictions, relative to other classifiers. ^25^ By mathematically defining MARS ‘ShineThrough’ and ‘Occlusion’ scores, we demonstrate how these metrics assess model performance as a function of the model’s ability to exclusively capture unique true positives not found by the other classifiers (‘ShineThrough’) and the model’s inability to capture true positives found by one or more of the other classifiers (‘Occlusion’). These metrics, designed to complement widely used traditional and alternative measures, add another layer to classifier assessment, provide crucial insight that helps better distinguish and explain the behavior of the top n-ranked classifiers, and can be further extended to find optimal complementary classifier combinations (ensembles).

## Related work

**Figure 1.**
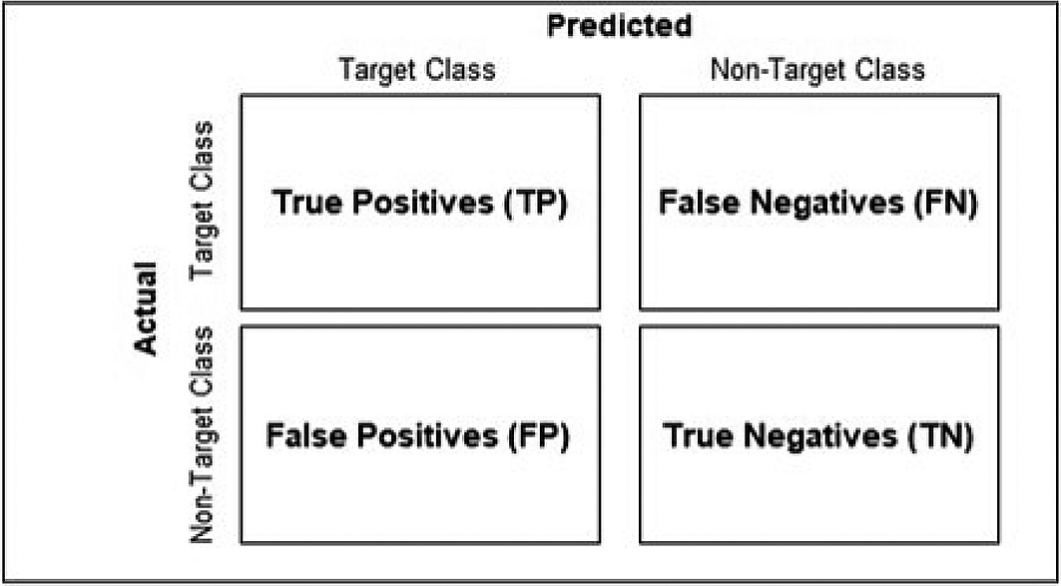
Format of a conventional classifier confusion matrix.

Binary classification Machine Learning (ML) performance metrics provide quantitative insight pertaining to different facets of a classifier’s true behavior, i.e., its performance on unseen data. For example, while precision is defined as the proportion of predicted positives that are actually positives, recall (sensitivity) is the overall proportion of positives that were correctly labelled as such. ^15^ These metrics, derived from the classifier’s confusion matrix ( Figure 1), offer complementary assessments concerning the classifier’s ability to detect and correctly label true positives, as evidenced by their mathematical definitions:

$$\text{Precision}=\frac{\mathrm{TP}}{\mathrm{TP}+\mathrm{FP}}$$

Abbreviations used: TP = True Positives, FP = False Positives.

$$\text{Recall}\;\left(\text{Sensitivity}\right)=\frac{\mathrm{TP}}{\mathrm{TP}+\mathrm{FN}}$$

Abbreviations used: FN = False Negatives.

Similar to sensitivity, which calculates the model’s true positive rate, specificity evaluates the overall proportion of negatives that were correctly labelled by the classifier (true negative rate). ^16^ Consequently, it follows a similar formulation:

$$\text{Specificity}=\frac{\mathrm{TN}}{\mathrm{TN}+\mathrm{FP}}$$

Abbreviations used: TN = True Negatives.

These metrics (precision, recall, specificity) provide crucial insight relating to classifier-class interactions. Other measures, such as accuracy and F score, ^17^ provide a more generalized interpretation of model behavior. F score, defined as the harmonic mean of precision and recall, evaluates the classifier’s performance across three confusion matrix components: TP, FP, FN, and can be defined as follows:

$$F_{\beta }=\left(1+\beta^{2}\right)\cdot \frac{\text{precision}\cdot \text{recall}}{\left(\beta^{2}\cdot \text{precision}\right)+\text{recall}}$$

Where β is arbitrarily chosen such that recall is β times as important as precision. The two most commonly used implementations are F _1_ and F _2_ scores. ^18^^–^^20^

Accuracy, unlike the aforementioned metrics, incorporates all four confusion matrix components into its calculations:

$$\text{Accuracy}=\frac{\mathrm{TP}+\mathrm{TN}}{\mathrm{TP}+\mathrm{TN}+\mathrm{FP}+\mathrm{FN}}$$

Unfortunately, accuracy is poor estimator of overall performance when the dataset labels are imbalanced, ^6^ as the classifier may be correctly labelling the majority class, thus, obtaining a high accuracy score, and misclassifying the minority class, at minimal accuracy cost. Regarding this, ^21^ proposed the use of the MCC ^12^ as a performance metric. MCC utilizes all four confusion matrix components, while also accounting for class imbalance. It does so by only generating a high score if both classes had the majority of their observations correctly predicted, regardless of class distribution. Similar to previous metrics, it is also derived from the classifier’s confusion matrix:

$$\mathrm{MCC}=\frac{\left(\mathrm{TP}\cdot \mathrm{TN}\right)-\left(\mathrm{FP}\cdot \mathrm{FN}\right)}{\sqrt{\left(\mathrm{TP}+\mathrm{FP}\right)\cdot \left(\mathrm{TP}+\mathrm{FN}\right)\cdot \left(\mathrm{TN}+\mathrm{FP}\right)\cdot \left(\mathrm{TN}+\mathrm{FN}\right)}}$$

MCC scores range from -1 to 1, representing perfect misclassification and classification, respectively. ^6^ As for visual metrics and evaluation of a classifier over multiple classification cut-off thresholds (ranked predictions), Receiver Operating Characteristics (ROC) curves ^22^^,^^23^ and Precision-Recall (PR) curves are generally considered to be the standard. ROC curves display what proportion of the total target class items were found by the classifier (sensitivity) in the x top- ranked target class predictions (x-axis). Comparing the classifier’s ROC curve against the benchmark 45-degree line, defined as the proportion of target class items found in a random sample of size x, allows the reader to rapidly determine whether the specific classifier is performing better than a random sample of size x would have been expected to. While the ROC curve does not provide a single-point estimate of the classifier’s performance, the ROC’s area under the curve (AUC) value does. ^22^ AUC scores, which range from 0 to 1, measure the classifier’s ability to distinguish between classes, and are often reported alongside the ROC curve. AUC values close to 1 indicate the classifier identified all, or almost all, of the available observations in the target class, as true positives, in its top-ranked observations (observations that classifier judged most likely to be in the target class).

Precision-Recall [PR] curves are sometimes used as an alternative to ROC curves, ^24^ to illustrate fluctuations in hit- and miss-rates, as increasing numbers of top-ranked observations are considered by a classifier. Notably, neither ROC curve nor PR curves indicate how many of the true positives in the top-ranked predictions are exclusive to the current classifier (i.e., were target-class items not found by any other classifier), nor how many of the false negatives are exclusive to the current classifier (i.e., were target-class items correctly found by any other classifier). Regarding this, the use of the MARS software artifact, proposed in Ref. 25, has been suggested as a way to overcome this limitation, which we further validate in this paper by presenting the mathematical foundations behind the software-level implementation of the MARS metrics.

## Methods

We assess overall classifier uniqueness across two separate dimensions: MARS ShineThrough and MARS Occlusion scores. These performance measures are briefly defined in Ref. 25 as:

1. MARS ShineThrough Score: The proportion of exclusive true positives discovered only by the classifier under consideration, relative to the total number of unique true positives (i.e., counting each target-class observation once only, if it is found by any classifier) discovered across all classifiers.
2. MARS Occlusion Score: The classifier’s proportion of exclusive false negatives (missed only by the current classifier) that were correctly labelled by at least one other classifier relative to the total number of unique true positives discovered across all classifiers (i.e., counting each target-class observation once only, if it is found by any classifier).

These performance measures are rigorously analyzed and mathematically anatomized in the subsections *MARS Shinethrough scores* and *MARS Occlusion scores* below.

### MARS ShineThrough Scores

Let *n* be the number of observations in a given dataset and *J* the set of classifiers, under consideration. Similarly, let *y _i_* be classifier’s predicted class label and *t _i_* the true class label (0 or 1) at observation *i.*

Then, we can define the total number of true positives (TTP _all_) as the sum, over *n* observations, of the maximum value of the product between predicted and true class labels across all *j* classifiers:

$${\mathrm{TTP}}_{\mathrm{all}}=\sum_{i}^{n}\max\left(y_{i,C_{j}}\cdot t_{i},\forall C_{j}\in J\right)$$ (1)

To determine the total number of exclusive true positives (ETP _C _*j*__) discovered by classifier *j*, i.e., target class observations found only by the current classifier and not found by the other classifiers, we use:

$${\mathrm{ETP}}_{C_{j}}=\sum_{i}^{n}\left(y_{i,C_{j}}\cdot t_{i}\right)-\max\left(y_{i,C_{j}}\cdot t_{i},\forall \in J\neq C_{j}\right)\cdot \mathrm{Ζi}\;$$ (2)

Where we sum (over *n* observations) the difference between the product of predicted and actual class labels and the maximum value of the same product across the remaining *j* -1 classifiers. Additionally, we multiply the latter by constant $Z_{i}$ , defined as:

Ζi=1⟺yi,Cj=1,ti=10,otherwise (2.1)

Consequently, the sum at observation *i* will have a non-zero value if and only if the classifier’s predicted and actual labels belong to the target class.

Then, using (1) and (2), we calculate the ShineThrough Score for classifier *j* as follows:

$${\text{ShineThrough}}_{C_{j}}=\frac{{\mathrm{ETP}}_{C_{j}}}{{\mathrm{TTP}}_{\mathrm{all}}}\;$$ (3)

Hence, MARS ShineThrough provides a much-needed numerical interpretation of the classifier’s comparative uniqueness, i.e., what proportion of the total number of true positives were exclusively identified by the classifier under consideration, relative to the competing classifiers. Occlusion scores, on the other hand, provide insight relating to the classifier’s comparative weaknesses.

### MARS occlusion scores

We define the total number of false negatives (EFN _C _*j*__) labelled by classifier *j* and correctly labelled by any of the remaining

*j −* 1 classifiers as:

$${\mathrm{EFN}}_{C_{j}}=\sum_{i}^{n}\max\left(y_{i,j,C_{j}}\cdot t_{i},\forall \in J\neq C_{j}\right)\cdot Ζ$$ (4)

Where, similar to Eq. (2), we find the maximum value of $y_{i,j}\cdot t_{i}\;$ across the remaining *j* − 1 classifiers and multiply the output by binary constant *Z _i_,* defined as:

Ζi=1⟺yi,Cj=0,ti=10,otherwise (4.1)

Thus, the summation will have a non-zero value at observation *i* if and only if the classifier under consideration incorrectly labelled the target class. Using (1) and (4), we then define the MARS Occlusion score for classifier *j* as:

$${\text{Occlusion}}_{C_{j}}=\frac{{\mathrm{EFN}}_{C_{j}}}{{\mathrm{TTP}}_{\mathrm{all}}}$$ (5)

Where we divide ${\mathrm{EFN}}_{C_{j}}$ by ${\mathrm{TTP}}_{\mathrm{all}}$ to determine what proportion of the classifier’s false negatives are true positives in any of the remaining *j* *–* 1 classifiers, therefore, quantitatively assessing the classifier’s comparative weaknesses.

### Notation reference

Table 1 provides a quick-reference glossary of the symbols used in our definitions.

**Table 1.** Glossary of symbols used.

Symbol	Definition
***i***	Observation number
***j***	Classifier number
***n***	Total number of observations
***y***_***i***, **C**_***j***__	Predicted class label for observation *i*, predicted by classifier *j*
***t***_***i***_	True class label for observation *i*
***J***	Set of classifiers
**C**_***j***_	Classifier *j*
***Z***_***i***_	Constant defined in (2.1) and (4.1) for observation *i*.
**TTP**_**all**_	Total number of unique true positives across all classifiers.
**ETP**_**C**_***j***__	Exclusive true positives found by classifier *j*.
**EFN**_**C**_***j***__	Exclusive false negatives for classifier *j*.

## Use cases

For the purposes of illustration, in the following subsections, we provide a stylized dataset and step-by-step, worked examples showing the computation of the MARS ShineThrough and MARS Occlusion scores, as well as the plotting of multiple MARS scores visually, in MARS charts. ^26^

While we provide an arbitrary, stylized dataset in this paper (to facilitate the understanding of the step-by-step examples), MARS metric performance on a real dataset can be found in Ref. 25. However, the latter does not provide any worked-out examples or rigorous mathematical explanations beyond the software-artifact’s outputs.

### Dataset

We created a simple, binary classification dataset with ten observations, each assigned an artificially generated “true” class label, for illustrative purposes. We also generated (predicted) labels for arbitrary classifiers: *J* = {C _1_, C _2_, C _3_, C _4_}. Actual (true) and classifier (predicted) labels can be seen in Table 2.

**Table 2.** Sample classifier prediction matrix.

		Observation ID, for Observation *i*									
		1	2	3	4	5	6	7	8	9	10
**Predicted**	**C**_1_	1	0	0	0	1	1	1	1	0	0
**class (C**_**1** *−* **4**_ **)**	**C**_2_	1	1	1	1	0	0	0	0	1	0
	**C**_3_	0	1	0	0	1	0	0	0	1	0
	**C**_4_	0	1	1	1	0	0	1	0	0	1
	**Actual class**	0	1	0	1	0	1	1	1	0	1

### MARS ShineThrough score metric: example computation

In order to calculate MARS scores, we first determine the total number of true positives discovered across all four classifiers using Eq. (1), that is:

$${\mathrm{TTP}}_{\mathrm{all}}=\sum_{i=1}^{10}\max\left(y_{i,C_{j}}\cdot t_{i},\forall C_{j}\in J\right)$$

We illustrate the sum’s inner calculations for the first two observations below:

@i=1,true class=0:

$$\max\;\left(1\times 0,1\times 0,0\times 0,0\times 0\right)=0$$

@i=2,true class=1:

$$\max\;\left(0\times 1,1\times 1,1\times 1,1\times 1\right)=1$$

Thus, the sum at *i* = 10 would be:

$${\mathrm{TTP}}_{\mathrm{all}}=\sum_{=1}^{10}0+1+0+1+0+1+1+1+0+1=6$$

Summing over all ten observations yields the value of 6, indicating that every target-class observation was correctly labelled by at least one classifier. This can be double-checked by looking at the classifiers’ target class predictions in Table 2 ( *i* = 2,4,6,7,8,10).

To calculate individual ShineThrough scores for the classifier under consideration, we divide the total number of exclusive true positives found by C *_j_* by the total number of unique true positives (i.e., correctly classified observations in the target-class) across all classifiers ( Eq. (3)). We demonstrate the procedure using C _1_:

Finally, we use Eq. (3) to obtain C _1_ ShineThrough scores:

$${\text{ShineThrough}}_{C_{1}}=\frac{2}{6}$$

This reveals that C _1_ alone accounts for one third of the discovered target class observations, suggesting its behavior is fairly unique amongst its peers. The calculations can be easily verified by looking at observations *i* = 6 and *i* = 8 in Table 2. Additionally, we can also calculate combined ShineThrough scores for two or more classifiers by merging their predictions and discarding their individual labels, prioritizing correct labels when possible ( Table 4).

For example, using Table 2 and Table 4, we can obtain the combined ShineThrough score for C _1_ and C _4_ using Eq. (1), (2), and (3), as follows:

$${\mathrm{ETP}}_{C_{1,4}}=\sum_{i=1}^{10}\left(y_{i,C_{1,4}}\cdot t_{i}\right)-\max\left(y_{i,C_{j}}\cdot t_{i},\forall \in J\neq C_{1,4}\right)\cdot Z_{i}$$

@i=10:

$${\mathrm{ETP}}_{C_{1,4}}=\sum_{i=1}^{10}0+0+0+0+0+1+1+1+0+1=4$$

$${\text{ShineThrough}}_{C_{1,4}}=\frac{4}{6}=\frac{2}{3}$$

This combined-ShineThrough indicates that two-thirds of the total target class observations Eq. (6), were exclusively discovered by classifiers C _1_ and C _4_, in combination, indicating that when combined, the classifiers perform extremely well relative to the remaining classifiers. Note that originally (prior to combining classifiers), the observation at *i* = 7 was not considered to be exclusive for any of the classifiers, however, once C _1_ and C _4_ had their predictions combined, it became exclusive for C _1,4_.

### MARS occlusion score metric: example computation

As for occlusions scores, we can calculate the total number of exclusive false negatives (missed only by the current classifier) that were correctly classified by any of the other classifiers following Eq. (4):

$${\mathrm{EFN}}_{C_{j}}=\sum_{i}^{n}\max\left(y_{i,C_{j}}\cdot t_{i},\forall \in J\neq C_{j}\right)\cdot Z_{i}$$

In the case of C _1_, the first two iterations of the sum are as follows:

@i=1:

$$y=1,t_{1}=0,k=0$$

$$\max\;\left(0\times 1,0\times 0,0\times 0\right)\times 0=0$$

@i=2:

$$y=0,t_{2}=1,k=1:$$

$$\max\;\left(1\times 1,1\times 1,1\times 1\right)\times 1=1$$

Following the same procedure, the final sum at *i* = 10 would be:

$${\mathrm{EFN}}_{C_{1}}=\sum_{i=1}^{10}0+1+0+1+0+0+0+0+0+1=3$$

Then, we calculate the Occlusion score for classifier C _1_ using Eq. (5):

$${\text{Occlusion}}_{C_{1}}=\frac{{\mathrm{EFN}}_{C_{1}}}{{\mathrm{TTP}}_{\mathrm{all}}}=\frac{3}{6}=\frac{1}{2}$$

Unlike ShineThrough scores (where higher scores suggest better performance), with Occlusion scores it is the case that lower scores suggest better performance. In the case of C _1_, its Occlusion score reveals that 50% of the target class observations discovered by any of the other competing classifiers, are being misclassified by C _1_ and correctly classified by at least one of the remaining classifiers. Similar to ShineThrough scores, we can also merge classifier predictions to calculate combined Occlusion scores. For example, for C _3_ and C _4_, whose combined predictions only have false negatives correctly labelled by the other classifiers (C _1_ or C _2_) at observations *i* = 6 and *i* = 8 ( Tables 1 and 3), we can calculate combined Occlusion _3,4_ as follows:

@i=6:

$$y=0,t_{6}=1,k=1$$

$$\max\;\left(1\times 1,0\times 1\right)\times 1=1$$

@i=8:

$$y=0,t_{8}=1,k=1$$

$$\max\;\left(1\times 1,0\times 1\right)\times 1=1$$

**Table 3.** Sample ShineThrough calculations for C _1._ *Z*_*i*_, constant defined for observation *i*.

Observation ( *i*)	Pred. class ( *y* *_i_*)	True class ( *t* *_i_*)	*Z* *_i_*	Inner sum - Eq. (2)
*1*	1	0	1	(1 × 0) − max (1 × 0, 0 × 0, 0 × 0) × 0 = **0**
*2*	0	1	0	(0 × 1) − max (1 × 1, 1 × 1, 1 × 1) × 0 = **0**
*3*	0	0	0	(0 × 0) − max (1 × 0, 0 × 0, 1 × 0) × 0 = **0**
*4*	0	1	0	(0 × 1) − max (1 × 1, 0 × 1, 1 × 1) × 0 = **0**
*5*	1	0	0	(1 × 0) − max (0 × 0, 1 × 0, 0 × 0) × 0 = **0**
*6*	1	1	1	(1 × 1) − max (0 × 1, 0 × 1, 0 × 1) × 1 = **1**
*7*	1	1	1	(1 × 1) − max (0 × 1, 0 × 1, 1 × 1) × 1 = **0**
*8*	1	1	1	(1 × 1) − max (0 × 1, 0 × 1, 0 × 1) × 1 = **1**
*9*	0	0	0	(0 × 0) − max (1 × 0, 1 × 0, 0 × 1) × 0 = **0**
*10*	0	1	0	(0 × 1) − max (0 × 1, 0 × 1, 1 × 1) × 0 = **0**

Then,

$${\text{Occlusion}}_{C_{3,4}}=\frac{1+1}{6}=\frac{1}{3}$$

Occlusion scores for the combined classifier, C _3,4_, indicate that one third of the target class labels were misclassified by the combination of classifier C _3_ and classifier C _4_, but correctly labelled by at least one of the remaining *j −* 1 classifiers.

### MARS charts

**Figure 2.**
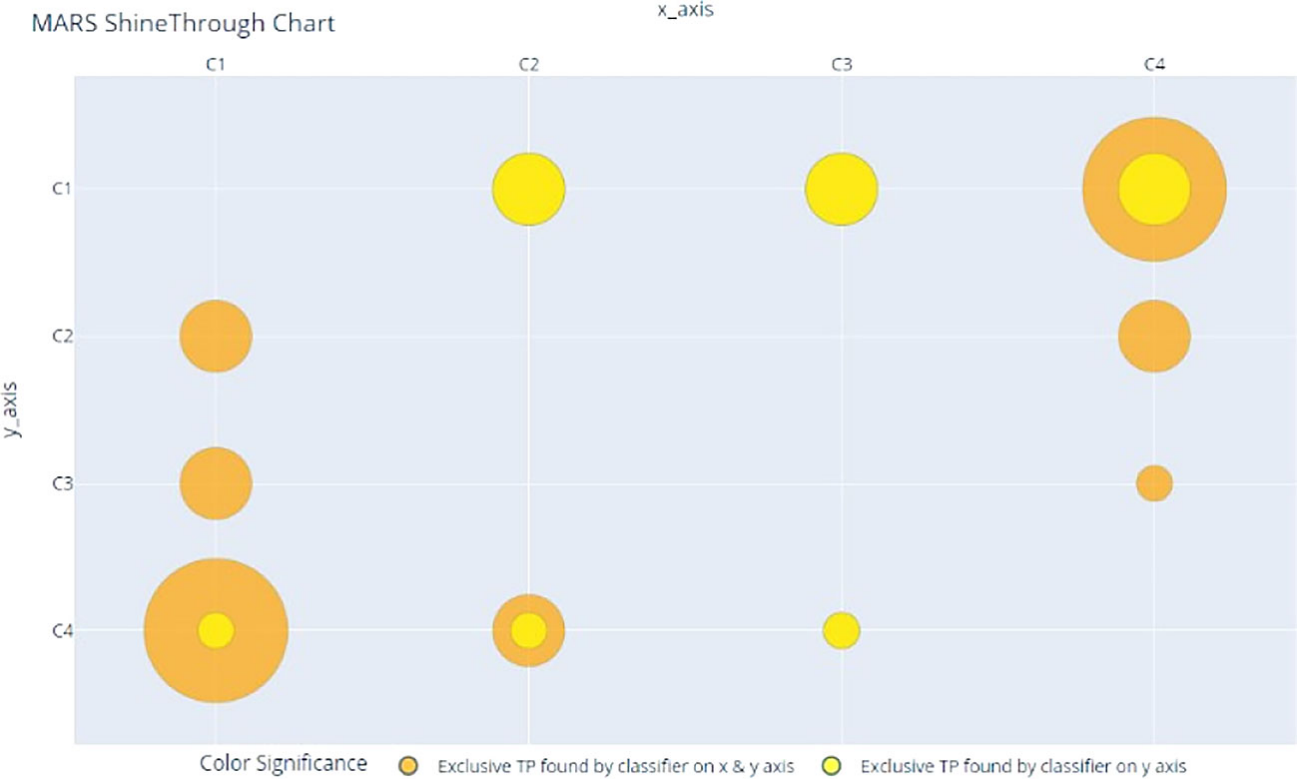
MARS ShineThrough Chart, comparing count (represented by bubble radius) of target-class observations (True Positives) exclusively spotted by classifiers C1 and the pairwise classifier combinations.

**Figure 3.**
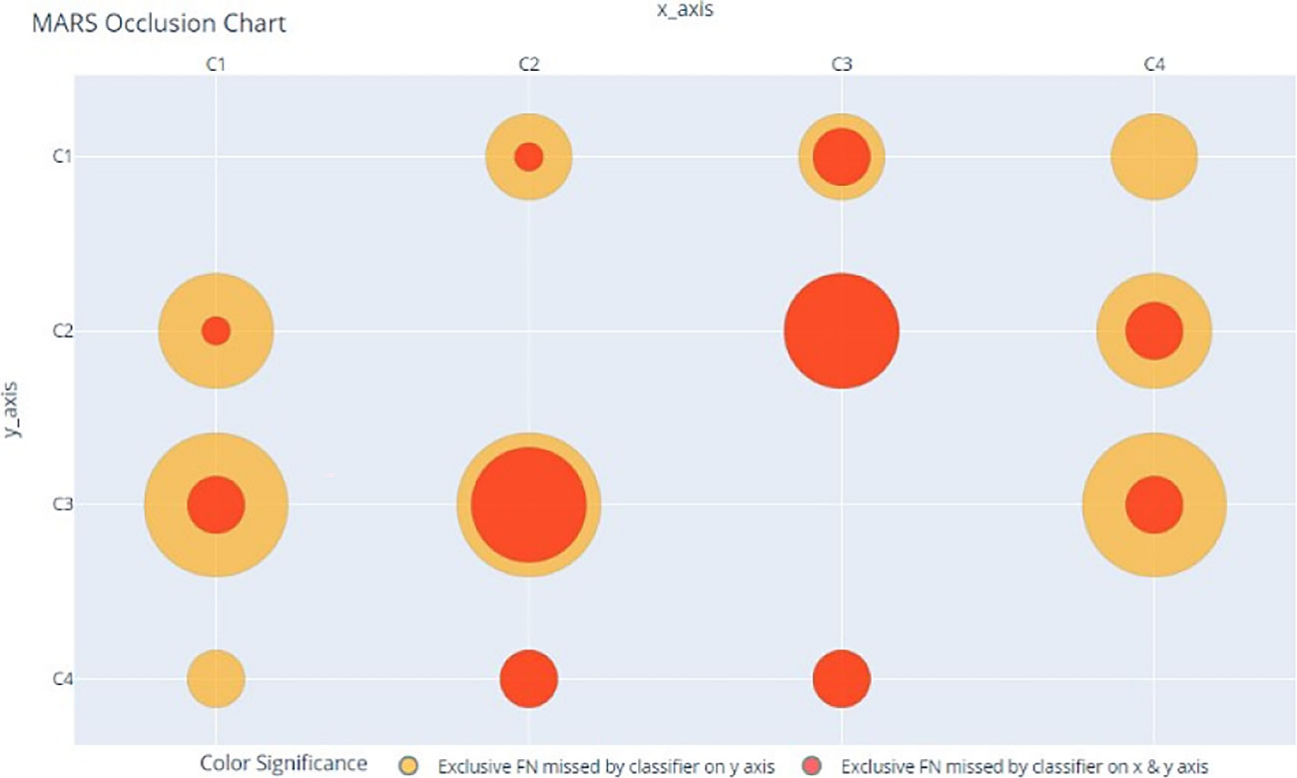
MARS Occlusion Chart, comparing count (represented by bubble radius) of target-class observations (False Negatives) exclusively missed by classifiers C1-4 and the pairwise classifier combinations.

MARS ShineThrough and Occlusion scores can also be visualized, allowing for the rapid depiction of the classifiers’ relative uniqueness. For our example dataset and classifiers above, the MARS metrics can be transformed from proportions (of total true positives) to counts (of unique hits or misses), and visualized, across individual and combined classifiers, as seen in Figure 2 and Figure 3, using a bubble-chart style format. Figure 2 is the MARS ShineThrough chart for classifiers C _1__-__4_; the radius of the yellow circle represents the number (count) of exclusive true positives found by the classifier on the y-axis. The radius of the orange circle represents the number of exclusive true positives found by both the classifier on the y-axis and x-axis, i.e., combined ShineThrough. Figure 3 is the MARS Occlusion chart: the radius of the red circle represents the number (count) of exclusive false negatives labelled by the classifier on the y-axis and the radius of the orange circle represents the combined number of exclusive false negatives labelled by the classifiers on both the x and y-axis.

Note that orange circles can only be as small as their respective yellow or red counterparts, which in turn may be as small as zero (indicating that the classifier found no exclusive true positives or false negatives).

## Discussion

Conventional metrics ( Table 5; columns 2-4) all points towards C _4_ being the unquestionably strongest classifier, due to its high accuracy (column 2), precision (column 3), and recall (column 4) values. However, MARS ShineThrough (ST) and Occlusion (OCC) scores ( Table 5; columns 5 and 6, respectively) and MARS charts ( Figure 2 and Figure 3) suggest that there is further room for improvement: Table 5 (ST column, row 1), and Figure 2 reveal that C _1_ is uniquely adept at spotting one third (0.33) of the target class items, and, while C _4_ performs reasonably well on its own ( Table 5; row 4), its combination with C _1_ results in the creation of a stronger classifier that accounts for two thirds (0.66) of the discovered target class items ( Table 5; ST column, row 5). Furthermore (see Table 5; OCC column, row 5; or see Figure 3), the combined classifier C _1,4_ has an Occlusion score of 0 (indicating that, if any target observations were missed by this classifier-combination, they were also missed by all other classifiers).

**Table 4.** Combined classifier prediction matrix.

		Observation ID, for Observation *i*									
		1	2	3	4	5	6	7	8	9	10
**Predicted**	**C**_1,4_	1	0	0	0	1	1	1	1	0	0
**Class**	**C**_2,3_	1	1	1	1	0	0	0	0	1	0
	**Actual class**	0	1	0	1	0	1	1	1	0	1

**Table 5.** Traditional vs MARS Metrics for the worked example.

Classifier	Metrics				
	Accuracy	Precision	Recall	ST	OCC
**C**_1_	0.50	0.60	0.50	**0.33**	0.50
**C**_2_	0.20	0.40	0.33	0.0	0.66
**C**_3_	0.30	0.33	0.16	0.0	0.83
**C**_4_	**0.70**	**0.80**	**0.66**	0.16	**0.33**
**C**_1,4_	**1.0**	**1.0**	**1.0**	**0.66**	**0.0**
**C**_3,4_	0.80	1.0	0.66	0.16	0.33

For brevity, we show only arbitrary selected classifier combinations here, rather than all possible classifier combinations. The best performing individual, and combined, classifier, on each metric, is shown with cell **bolded**.

While some classifier combinations may improve overall performance, the opposite is also possible. For example, Figure 3 shows that the combination of C _3_ and C _4_ produces MARS scores identical to those of C _4_ alone, indicating that it is a weak combination, and should, therefore, be avoided. While traditional performance metrics gauge individual classifier capabilities by quantitively interpreting classifier-data interactions, MARS scores and charts measure classifier capabilities by simultaneously interpreting both classifier-data and classifier-classifier interactions.

## Conclusions

In this paper, we presented the mathematical background and interpretation for two novel binary classification performance metrics – MARS ShineThrough and MARS Occlusion scores, whose software-level implementation, in the Python language, was recently described in Ref. 25. The formal definition of the MARS method, provided in this paper, will allow the research community to verify the correctness of the MARS method (through peer-review), accurately implement the MARS method in other programming languages (such as JavaScript, PHP, and R), and develop novel alternatives to, and enhancements to, the MARS method (such as visualizations that chart MARS metrics across multiple classifier cut-off thresholds instead of the single classifier cut-off threshold illustrated here). The stylized dataset and worked sample calculations provided in the Use cases section of this paper, above, is usable by the research community as a test case, to validate the correctness of each computational step of future software implementations. MARS metrics and MARS charts add yet another layer to the process of classifier assessment, providing crucial insight about each classifier’s behavior relative to that of its peers. ShineThrough scores evaluate the comparative unique strengths of the classifier, by determining the proportion of total true positives that were exclusively found by the classifier. On the other hand, Occlusion scores measure the proportion of observations that were correctly labelled by the other classifiers but misclassified by the current classifier, i.e., the classifier’s comparative unique weaknesses.

Naturally, the metrics synergize well with conventional measures, as the latter are constrained to the individual classifier’s confusion matrix, severely limiting the breadth of their analysis, while the former make use of the entire observation sample space, thus, evaluating classifier behavior from a previously unseen standpoint: number of target class observations spotted or missed only (i.e., exclusively) by one classifier. This was demonstrated throughout the provided worked-out examples, which calculated ShineThrough and Occlusion scores for our stylized dataset ( Tables 2 and 4), and in Ref. 25 with a real dataset, albeit without the comprehensive mathematical explanation and examples presented in this paper. As a result, the MARS methodological framework adds a new classifier-comparison dimension – exclusive hits and misses – not expounded by conventional classifier evaluation methods.

## Data availability

All data underlying the results are available as part of the article and no additional source data are required.

## Software availability

Webapp: https://mars-classifier-evaluation.herokuapp.com

Source code available from: https://github.com/SoftwareImpacts/SIMPAC-2021-191

Archived source code at time of publication: https://doi.org/10.24433/CO.2485385.v1 ^26^

License: MIT
