# Supplementary material for: Formal definition of the MARS method for quantifying the unique target class discoveries of selected machine classifiers
Source: F1000Res. 2022 Jul 1;11:391. Originally published 2022 Apr 4. [Version 2] doi: 10.12688/f1000research.110567.2 (PMC9350436; doi:10.12688/f1000research.110567.2)
Supplement: Peer review history

# Peer review: Reviewer response for version 2

**DOI:** 10.5256/f1000research.135201.r142931

**Published:** 2022-08-03

**Authors:** Samir Chatterjee

**Affiliations:** 1 School of Information and Technology Management, Claremont Graduate University, Claremont, USA

I am happy with the changes.

Is the rationale for developing the new method (or application) clearly explained?

Partly

Is the description of the method technically sound?

Yes

Are the conclusions about the method and its performance adequately supported by the findings presented in the article?

Partly

If any results are presented, are all the source data underlying the results available to ensure full reproducibility?

Yes

Are sufficient details provided to allow replication of the method development and its use by others?

Yes

Reviewer Expertise:

ML in Healthcare

I confirm that I have read this submission and believe that I have an appropriate level of expertise to confirm that it is of an acceptable scientific standard.

# Peer review: Reviewer response for version 2

**DOI:** 10.5256/f1000research.135201.r142932

**Published:** 2022-07-18

**Authors:** Timothy A. Warner

**Affiliations:** 1 Department of Geology and Geography, West Virginia University, Morgantown, WV, USA

Many thanks for the careful revisions and particularly for the detailed response to my suggestions. The revised paper makes a valuable contribution.

 Some very minor, final suggestions are listed below:

1. The MARS acronym in the abstract (p1) and the text (p3) seem to be different: the latter seems to be missing “Specificity” at the end.
2. In the abstract, I suggest adding “may” in front of “share nearly identical performance” (because classifier performance is sometimes not nearly identical).
3. In the second sentence of the last paragraph on p3, I suggest adding “Ref.” or something similar to make the sentence easier to read. (E.g., so that it would read, “Recently, Ref. 1 evaluated…”).
4. P6, in the paragraph below the two numbered paragraphs: I suggest replacing “calculations are identical” with “calculations are similar”.
5. Equation 2. Shouldn’t the variable *i* following *Z* be a subscript?

Is the rationale for developing the new method (or application) clearly explained?

Partly

Is the description of the method technically sound?

Partly

Are the conclusions about the method and its performance adequately supported by the findings presented in the article?

Partly

If any results are presented, are all the source data underlying the results available to ensure full reproducibility?

Yes

Are sufficient details provided to allow replication of the method development and its use by others?

Yes

Reviewer Expertise:

Remote sensing

I confirm that I have read this submission and believe that I have an appropriate level of expertise to confirm that it is of an acceptable scientific standard.

# Peer review: Reviewer response for version 1

**DOI:** 10.5256/f1000research.122189.r136525

**Published:** 2022-06-16

**Authors:** Samir Chatterjee

**Affiliations:** 1 School of Information and Technology Management, Claremont Graduate University, Claremont, USA

The paper proposes two new binary classifier metrics in addition to existing traditional metrics such as accuracy, precision, recall, F-score. Two classifiers of equal accuracy may each have the unique ability to identify distinct observations from the target class.

 MARS ShineThrough and MARS Occlusion scores are mathematically presented.

 On page 7, where MARS occlusion scores is first defined, it should read "total number of expected false negatives (EFNcj)."

 In ML, combining algorithms is not too common. While ensemble methods use a similar core algorithms but creates different variations of the classifier (many trees in a Random Forest implementation), it may not be practically feasible to combine classifiers that are inherently built using different algorithms (Logistic regression, SVM, KNN). In those cases, how will this technique apply?

 The visual bubble graphs are also little hard to understand.

 When comparing between different classifiers, while ST and OCC may tell us unique distinguishing capability of the classifiers, it is also important to have a discussion of the consequences of the TP and FN especially when it might be related to disease predictions. What are the trade-offs?

Is the rationale for developing the new method (or application) clearly explained?

Partly

Is the description of the method technically sound?

Yes

Are the conclusions about the method and its performance adequately supported by the findings presented in the article?

Partly

If any results are presented, are all the source data underlying the results available to ensure full reproducibility?

Yes

Are sufficient details provided to allow replication of the method development and its use by others?

Yes

Reviewer Expertise:

ML in Healthcare

I confirm that I have read this submission and believe that I have an appropriate level of expertise to confirm that it is of an acceptable scientific standard, however I have significant reservations, as outlined above.

# Response

**Published:** 2022-06-17

**Authors:** Peter Ractham

**Affiliations:** Thammasat Business School, Thailand

**Manuscript Number: 110567**

 Dear Dr. Chatterjee,

 Thank you for your helpful comments and suggestions regarding our manuscript, *Formal definition of the MARS method for quantifying the unique target class discoveries of selected machine classifiers*. We have edited the manuscript with the corrections and clarifications prompted by your suggestions.

 Below this letter, we have addressed the comments in a point-by-point manner. Our response, **bolded**, includes a brief explanation behind the initial reasoning and how and where the paper was modified.

 Thank you for your time and consideration.

 Sincerely,

 Felipe Restrepo

 Namrata Mali

 Alan Abrahams

 Peter Ractham

**Comments to the Authors:**

1. On page 7, where MARS occlusion scores is first defined, it should read "total number of expected false negatives (EFN _Cj_)

**Thank you for noticing this. We have made this fix.**

1. In ML, combining algorithms is not too common… it may not be practically feasible to combine classifiers that are inherently built using different algorithms (Logistic regression, SVM, KNN). In those cases, how will this technique apply? 

**We have clarified in the manuscript that we are not proposing that the algorithm procedures be combined, but rather that the predicted positive observations of the algorithms be combined.  Specifically, the intersection of the sets of predicted positives of the algorithms is taken, which is a straightforward operation.  The combination of predicted positives, from two algorithms that each suggest distinct true positives, allows the data scientist to efficiently boost the number of true positives while constraining total positive predictions, which is highly desirable for applications like our prototypical application: maximizing the volume of safety concerns found in online reviews, while minimizing the close-reading verification effort required to determine if a predicted positive is a true positive.**

1. The visual bubble graphs are also little hard to understand.

**Thank you for your suggestion. To assist readers who find bubble charts difficult to interpret, we have supplemented the visual bubble graphs with MARS bar charts, which show the total distinct true positives, for each algorithm, sorted from algorithm with most distinct true positives down to the algorithm with the least distinct true positives. **

1. It is also important to have a discussion of the consequences of the TP and FN especially when it might be related to disease predictions. What are the trade-offs?

**Thank you for pointing out the importance of adding this discussion.  We have added the following: “The MARS evaluation mechanism was developed for a prototypical application of maximizing the volume of safety concerns found in online reviews, while constraining the close-reading verification effort required to determine if predicted positives are true positive.  That is, the MARS method assists with elevating binary classifier yield: that is, increasing verified true positives per unit of effort reviewing predicted positives.  The MARS evaluation mechanism is best suited to applications where the false positive cost is low, such as our prototypical application of discovering safety concerns in online reviews: a true positive (online review that contains a safety concern) is valuable, while a false positive (online review that does not contain a safety concern) has low cost, as each false positives wastes only a little reading effort, especially when there are few online reviews (predicted positives) shortlisted by the ML algorithm(s) for escalated attention by a human reviewer who is manually reviewing the predicted positive observations.  For other applications – such as disease discovery – where the false positives, and false negatives, have differing trade-offs, the MARS evaluation method presented here may not be appropriate, and an inverted MARS evaluation method, aimed at maximizing true negatives, may be preferable.**

**Thank you again for your helpful observations and suggestions, which have helped us improve the clarity of the manuscript.**

# Response

**Published:** 2022-06-17

**Authors:** Peter Ractham

**Affiliations:** Thammasat Business School, Thailand

Dear Reviewer,

 It seems that we'll have to wait for the update version of the manuscript from F1000 before we can submit the revised manuscript suggested by you. We'll update it as soon as possible. Thank you.

# Peer review: Reviewer response for version 1

**DOI:** 10.5256/f1000research.122189.r136529

**Published:** 2022-05-17

**Authors:** Timothy A. Warner

**Affiliations:** 1 Department of Geology and Geography, West Virginia University, Morgantown, WV, USA

This paper describes statistics that summarize similarity of the labelling of unknown samples by different classifiers. The method has two levels: (1) individual classifiers vs the rest and (2) groups of two classifiers vs the rest. The method and the visualization of the results were described in an earlier paper; this paper provides a more thorough definition of the statistics and a worked, hypothetical example. 

 The fact this paper has the express aim of providing clarification to an earlier paper may constrain how open the authors are to modifying their approach.

 Major suggestions

1. The two measures of shine through and occlusion are described numerous times as respectively measures of exclusive true positives and exclusive false negatives. However, the definition of exclusive seems to differ in the two cases. For shine through, exclusive means true positives (TP) for *only* the classifier of interest, and no other classifier (i.e. FN for all other classifiers). For occlusion exclusive means false negatives (FN) for the classifier of interest, but a TP for *any* (i.e., at least one) other classifier. Thus for occlusion the word “exclusive” has a more relaxed meaning than for shine through. If the meaning of exclusive for occlusion were the same as for shine through, then the definition of occlusion would be a FN for the classifier of interest, and no other classifier. 
2. The paper focuses on “exclusive” TP and FP. However, as Figure 1 from the paper shows, there are two other types of classification outcomes: false positives (FP) and true negatives (TN). A key aspect of classifier behavior is the trade-off between FN and FP. Is there any reason for not developing similar metrics for FP and TN, and thus providing a comprehensive, instead of partial, view of the differences between classifiers? 
3. The paper makes a valuable contribution in providing statistical measures that compare decision boundaries between classifiers. However, I think it is potentially confusing to suggest that the MARS statistics are “alternative classification performance metrics” that overcome “limitations” of “traditional performance metrics.” I think that is a bit like saying the problem with the mean as a measure of central tendency is that it doesn’t measure autocorrelation. The MARS statistics don’t seem to be performance metrics, in the sense of quantifying accuracy. I think being clear about the purpose and role of MARS statistics is important in helping readers understand what information the MARS can offer.
4. Based on the above, I suggest removing the extensive discussion of Kappa, F-score, MCC, AUC, ROC, PR curves, and class imbalance, which seems to be a distraction. Furthermore, it seems to me class imbalance will affect MARS metrics as much as any other statistic, so I don’t follow the argument that MARS represents a method to address this limitation in conventional accuracy statistics. I think all you need to say is that two classifiers can have the same summary accuracy statistics (such as overall accuracy, precision and recall), but have different decision boundaries. MARS helps one explore those differences. Similarly, in the discussion, I suggest emphasizing that the power of the MARS measures is not in clarifying the accuracy of the various classifiers, but rather in highlighting differences in their decision boundaries.
5. For the MARS charts, I suggest following the example of a covariance matrix (where the variance is on the diagonal, and covariance on off-diagonal positions), and place the single classifier values on the diagonal, and the classifier combinations on the off-diagonal positions. (However, I suggest keeping the different colors, which I found useful.) I think the use of the diagonal and off-diagonal in this way is conceptually clearer, and also has the benefit that you don’t have the problem of which one to prioritize when the two circles have the same diameter.
6. I don’t understand Table 4. The numbers in table 4 for C1,4 seem to be a duplicate of C1 in Table 2, and C2,3 a duplicate of C2.  Crucially, I can’t relate it to the calculation of ETPsub(C1,4), nor does it seem to agree with table 5. 
7. In table 5, I don’t understand how the overall accuracy, precision and recall for the combination of classifiers (C1,4 and C3,4) are calculated. For example, I don’t see what objective rule combining C1 and C4 could result in class labels that indicate 100% accuracy for these samples. 

Minor suggestions

1. Perhaps explain the MARS acronym? I don’t understand the reference to sensitivity and specificity in the context of shine through and occlusion (especially since specificity is the TN as a proportion of the reference Negative class; the current MARS statistics do not seem to include an “exclusive” TN measure).
2. I suggest defining MARS acronym in the main body – currently it seems to be only defined in the abstract. (Unless I missed it. If so, sorry.)
3. Second sentence under the heading “Related work” – I suggest that in defining the recall, add the word “actual” prior to “positives”, so that the definition becomes “the overall proportion of *actual* positives that were correctly labelled as such.”
4. “Accuracy” – defined on p5. I suggest using the term “overall accuracy” rather than just “accuracy” to differentiate this from the generic concept of accuracy.
5. I think the equations would be easier to follow if you moved the reference to Table 1 to the start of the section with the equations.
6. I suggest not using the same symbol for more than one purpose. For example, the constant Z-sub-i has different definitions in 2.1 and 4.1. (When I first read the paper, I incorrectly used the 2.1 definition when I was working through the occlusion example. Using a different letter for the 2 constants would avoid this problem.)
7. Similarly, in eqn 2, it was a bit confusing to me that subscript j on the left side of the equation could (in fact, has to) simultaneously represent a different value on the right hand side of the equation. Using a different symbol on the left side will obviate this confusion.
8. P8 – Second-last paragraph “To calculate individual Shine through scores”….I suggest referring to Table 3 here to clarify how ETP is calculated.
9. P9 –Has variable k been defined? Is it perhaps Z-sub-i?
10. P9. The example of occlusion for C1, @i=1. In the first worked example, is the first 0 and 1 (y11 and t1) switched? I.e, it seems to me that this should read “max (1 x 0, 0 x 0, 0 x 0) x 0 = 0”?
11. Table 3. The value for Z-sub-i for observation 1 is listed in the table as 1. Should it not be 0?
12. End of first paragraph below Table 3. The reference to “Tables 1 and 3” – shouldn’t this be to “Tables 1 and 4”?
13. MARS charts – the discussion and captions simplifies the MARS metrics as “counts” – but they are actually defined as proportions. I think adding a legend that indicates how circle size relates to proportions would be useful.

Is the rationale for developing the new method (or application) clearly explained?

Partly

Is the description of the method technically sound?

Partly

Are the conclusions about the method and its performance adequately supported by the findings presented in the article?

Partly

If any results are presented, are all the source data underlying the results available to ensure full reproducibility?

Yes

Are sufficient details provided to allow replication of the method development and its use by others?

Yes

Reviewer Expertise:

Remote sensing

I confirm that I have read this submission and believe that I have an appropriate level of expertise to confirm that it is of an acceptable scientific standard, however I have significant reservations, as outlined above.

# Response

**Published:** 2022-06-16

**Authors:** Peter Ractham

**Affiliations:** Thammasat Business School, Thailand

**Manuscript Number: 110567**

 Dear Dr. Warner,

 Thank you for reviewing our manuscript, *Formal definition of the MARS method for quantifying the unique target class discoveries of selected machine classifiers*. We greatly appreciate the careful review and valuable feedback you have provided. We have incorporated the suggested changes to the paper and software artifact.

 Below this letter, we have addressed the comments in a point-by-point manner. Our response, **bolded**, includes a brief explanation behind the initial reasoning and how and where the paper was modified.

 Thank you for your time and consideration.

 Sincerely,

 Felipe Restrepo

 Namrata Mali

 Alan Abrahams

 Peter Ractham

**Comments to the Authors:**

1. The two measures of shine through and occlusion are described numerous times as respectively measures of exclusive true positives and exclusive false negatives. However, the definition of exclusive seems to differ in the two cases. For shine through, exclusive means true positives (TP) for *only* the classifier of interest, and no other classifier (i.e. FN for all other classifiers). For occlusion exclusive means false negatives (FN) for the classifier of interest, but a TP for *any* (i.e., at least one) other classifier. Thus for occlusion the word “exclusive” has a more relaxed meaning than for shine through. If the meaning of exclusive for occlusion were the same as for shine through, then the definition of occlusion would be a FN for the classifier of interest, and no other classifier. 

**Thank you for pointing this out. We have modified the definition of occlusion so that the definition of exclusive true positives now directly parallels that of exclusive false negatives. The updated mathematical formulation is now:**

**Formula 1:**

*https://f1000researchdata.s3.amazonaws.com/linked/432866.formula1.png*

**Formula 2:**

*https://f1000researchdata.s3.amazonaws.com/linked/432867.formula2.png*

**Following this formulation, Occlusion scores now represent the proportion of the classifier’s unique misses, relative to discovered true positives. A high Occlusion score may suggest that the classifier has a rather specific struggle within the classification task, which the remaining models are capable of handling.**

**Within the manuscript, we updated the MARS Occlusion Scores section (p. 5) with the revised mathematical formulation, the MARS Occlusion Score Metric: Example Computation section (p. 8) with the revised mathematical formulation, and Table 4 (p.10) Occlusion scores. Figure 3 (MARS Occlusion Chart) in the MARS charts section (p. 9) was also updated to reflect the updated definition. **

1. The paper focuses on “exclusive” TP and FP. However, as Figure 1 from the paper shows, there are two other types of classification outcomes: false positives (FP) and true negatives (TN). A key aspect of classifier behavior is the trade-off between FN and FP. Is there any reason for not developing similar metrics for FP and TN, and thus providing a comprehensive, instead of partial, view of the differences between classifiers? 

**Thank you for pointing this out. Given that MARS metrics were designed as a tool to optimize big-data, machine-driven discovery efforts in which the value of TPs and the cost of FNs is high, - e.g., flagging potentially hazardous products via online reviews – we focused on defining MARS through TPs and FNs. The approach can easily be adapted to applications in which the value and cost of TNs/FPs is high.**

**Within the manuscript, we have clarified that the approach is easily replicated with TNs and FPs, but we omit detailed calculations (as they would be nearly identical to those of TPs and FNs) for brevity (Methods section, p. 4).  **

1. The paper makes a valuable contribution in providing statistical measures that compare decision boundaries between classifiers. However, I think it is potentially confusing to suggest that the MARS statistics are “alternative classification performance metrics” that overcome “limitations” of “traditional performance metrics.” I think that is a bit like saying the problem with the mean as a measure of central tendency is that it doesn’t measure autocorrelation. The MARS statistics don’t seem to be performance metrics, in the sense of quantifying accuracy. I think being clear about the purpose and role of MARS statistics is important in helping readers understand what information the MARS can offer.

**Thank you for your suggestion. We intended for MARS metrics to be used alongside traditional metrics as a tool to optimize big-data, machine-driven discovery efforts. MARS scores were designed to complement traditional ones (e.g., accuracy, recall, precision) in high-volume data applications, where models are likely to have similar conventional summary statistics, even if their decision boundaries are fundamentally different. In these cases, the depth of traditional metric analysis may be significantly limited, as results would simply suggest that all models employed worked well on the data, providing no differentiating power within the set of classifiers. Whereas using MARS metrics, which are far more likely to detect differences in classifier behavior, alongside traditional metrics, would allow for a more complete analysis to be made about the model’s overall (individual and comparative) performance.**

**Within the manuscript, we have modified the Introduction section (p. 2 – 3) so that the purpose and role of MARS statistics is clear for readers. **

1. Based on the above, I suggest removing the extensive discussion of Kappa, F-score, MCC, AUC, ROC, PR curves, and class imbalance, which seems to be a distraction. Furthermore, it seems to me class imbalance will affect MARS metrics as much as any other statistic, so I don’t follow the argument that MARS represents a method to address this limitation in conventional accuracy statistics. I think all you need to say is that two classifiers can have the same summary accuracy statistics (such as overall accuracy, precision and recall), but have different decision boundaries. MARS helps one explore those differences. Similarly, in the discussion, I suggest emphasizing that the power of the MARS measures is not in clarifying the accuracy of the various classifiers, but rather in highlighting differences in their decision boundaries.

**Thank you for your suggestion. The class imbalance discussion was meant to highlight the vulnerabilities of conventional metrics and bring forth the need for novel metrics capable of examining classifiers from a different standpoint. We did not intend to imply that MARS represents a method to address this limitation. Rather, we intended to express that conventional metrics would greatly benefit from the use of MARS alongside, as doing so would allow for a more objective and in-depth analysis of the model’s behavior – which we have now made clear in the Introduction section, rendering the class imbalance discussion unnecessary.**

**Within the manuscript, we have removed the class imbalance discussion in the Introduction section (p. 3) and significantly shortened the Related Works section by doing the same (p. 3-4). Within Related Works (p. 3-4), we also reduced the discussion pertaining to PR and ROC curves. The Discussion section (p. 10) was reworked to better emphasize the power of MARS metrics in spotting differences between classifier behavior and optimizing model combinations. **

1. For the MARS charts, I suggest following the example of a covariance matrix (where the variance is on the diagonal, and covariance on off-diagonal positions), and place the single classifier values on the diagonal, and the classifier combinations on the off-diagonal positions. (However, I suggest keeping the different colors, which I found useful.) I think the use of the diagonal and off-diagonal in this way is conceptually clearer, and also has the benefit that you don’t have the problem of which one to prioritize when the two circles have the same diameter.

**Thank you for your suggestion. We have implemented the suggested changes to the MARS charts and updated Figures 2 and 3 (Mars charts section, p. 9-10).**

1. I don’t understand Table 4. The numbers in table 4 for C1,4 seem to be a duplicate of C1 in Table 2, and C2,3 a duplicate of C2.  Crucially, I can’t relate it to the calculation of ETPsub(C1,4), nor does it seem to agree with table 5. 

**Thank you for pointing this out. We have removed Table 4 from the manuscript. Upon review, it does not align with the reworked Discussion and Introduction sections, as combined MARS metrics are meant to facilitate the discovery of classifiers with complementary decision boundaries; they are not designed for traditional classifier ensemble creation, as Table 4 suggested. **

1. In table 5, I don’t understand how the overall accuracy, precision and recall for the combination of classifiers (C1,4 and C3,4) are calculated. For example, I don’t see what objective rule combining C1 and C4 could result in class labels that indicate 100% accuracy for these samples. 

**Thank you for pointing this out. As per the previous suggestion, we re-evaluated Table 4 and determined it was best eliminated. Consequently, C1,4 and C3,4 have been removed from Table 5.**

1. Perhaps explain the MARS acronym? I don’t understand the reference to sensitivity and specificity in the context of shine through and occlusion (especially since specificity is the TN as a proportion of the reference Negative class; the current MARS statistics do not seem to include an “exclusive” TN measure).

**Thank you for your suggestion. Since sensitivity examines the model’s target-class detection capabilities with respect to the complete TP sample space, we referenced MARS as a tool to determine relative sensitivity, as it examines the model’s target-class detection capabilities with respect to competing classifiers, rather than the “ground truth”. We followed the same logic when referring to relative specificity. However, since we only briefly mention the ease of adaptability to TNs and FPs (refer to revision 2.), we have removed the specificity reference (Abstract, p. 1 & Introduction, last paragraph). **

1. I suggest defining MARS acronym in the main body – currently it seems to be only defined in the abstract. (Unless I missed it. If so, sorry.)

**Thank you for your suggestion. We had previously defined the MARS acronym in the main body (Introduction, last paragraph), and have now added an additional definition when first mentioned (Introduction, p. 2 ¶ 3).**

1. Second sentence under the heading “Related work” – I suggest that in defining the recall, add the word “actual” prior to “positives”, so that the definition becomes “the overall proportion of *actual* positives that were correctly labelled as such.”

**Thank you for your suggestion. We have added “actual” prior to positives (Related work, p. 3, ¶ 1). **

1. “Accuracy” – defined on p5. I suggest using the term “overall accuracy” rather than just “accuracy” to differentiate this from the generic concept of accuracy.

**Thank you for your suggestion. We have added “overall” prior to accuracy (Related Work, p. 4, ¶ 5).**

  

1. I think the equations would be easier to follow if you moved the reference to Table 1 to the start of the section with the equations.

**Thank you for your suggestion. We have moved Table 1 to the start of the Methods section (p. 5). **

1. I suggest not using the same symbol for more than one purpose. For example, the constant Z-sub-i has different definitions in 2.1 and 4.1. (When I first read the paper, I incorrectly used the 2.1 definition when I was working through the occlusion example. Using a different letter for the 2 constants would avoid this problem.)

**Thank you for your suggestion. We have changed the constant Z-sub-i to R-sub-i for the Occlusion metric. **

1. Similarly, in eqn 2, it was a bit confusing to me that subscript j on the left side of the equation could (in fact, has to) simultaneously represent a different value on the right hand side of the equation. Using a different symbol on the left side will obviate this confusion.

**Thank you for your suggestion. We have added C _w _which represents the classifier of interest and resolves the confusion. Within the manuscript, we updated equations 1 – 5 (Methods section, p. 5 – 6) to reflect this change. The new term was also added to Table 1 (p. 5). **

1. P8 – Second-last paragraph “To calculate individual Shine through scores”….I suggest referring to Table 3 here to clarify how ETP is calculated.

**Thank you for pointing this out. We have referenced Table 3 for the worked-out ETP calculation example (** **MARS ShineThrough score metric: example computation,**

**p. 7). **

1. P9 –Has variable k been defined? Is it perhaps Z-sub-i?

**Thank you for pointing this out. Yes, it was meant to be Z-sub-i (now R-sub-i for Occlusion scores). Within the manuscript we have substituted all mentions of k for R-sub-i (** **MARS occlusion score metric: example computation, p. 8 -9)**

1. P9. The example of occlusion for C1, @i=1. In the first worked example, is the first 0 and 1 (y11 and t1) switched? I.e, it seems to me that this should read “max (1 x 0, 0 x 0, 0 x 0) x 0 = 0”?

**Thank you for pointing this out. Yes, it should be ‘1 x 0’ instead of ‘0 x 1’. We have corrected the order within the manuscript (** **MARS occlusion score metric: example computation,** **p. 8). **

1. Table 3. The value for Z-sub-i for observation 1 is listed in the table as 1. Should it not be 0?

**Thank you for pointing this out. Yes, it should be zero. We have made the change in Table 3 (** **MARS ShineThrough score metric: example computation,** **p. 7). **

1. End of first paragraph below Table 3. The reference to “Tables 1 and 3” – shouldn’t this be to “Tables 1 and 4”?

**Thank you for pointing this out. The initial reference should have been Tables 1 and 4. However, since Table 4 was removed, it has been updated to ‘Table 1’  (** **MARS occlusion score metric: example computation,** **p. 9).**

1. MARS charts – the discussion and captions simplifies the MARS metrics as “counts” – but they are actually defined as proportions. I think adding a legend that indicates how circle size relates to proportions would be useful.

**Thank you for your suggestion. We have added an additional line to the captions of Figures 2 and 3 (Mars charts section, p. 8 – 9) explaining that bubble size is proportional to ShineThrough/Occlusion score: the larger the bubble, the higher the classifier(s) ShineThrough score.**
